# Working Women in Large Towns

**Published:** 1890-08-30

**Authors:** 


### Working Women in Large Towns.

### VIII.—SHIRT, SACK, AND UMBRELLA MAKERS.

### Shirt Makers.

Shirt-making seems to be the trade to which women naturally turn, when they suddenly find themselves called upon to earn, or partly earn, their own bread. They may be the wives of clerks, struggling to maintain a family of small children, and to keep up a decent appearance of respectability, on eighty pounds a-year; they may be ladies even, who have lost the whole or part of their slender income, and try in despair to make what they can of one of the "accomplishments" learnt in their happier days. Most of them are not wholly dependent upon their earnings; they have saved some portion from the wreck, or their friends will do something for them—perhaps even make up what they cannot earn themselves; and so it comes to pass that they will take work at almost any price, thus underselling those who must live upon what they earn, and to whom a docked-off halfpenny or penny per shirt means descent from a bare livelihood to hunger and misery. Leaving them and their (to some extent) skilled work, we come to the wife of the out-of-work labourer, or it may be his widow, who has little or no skill, and toils away at the common kinds of shirts, competing with girls and young women who, through lack of enterprise or money, have never been able to learn a trade, but have just taken to work which needs but a thimble and needle and the slenderest portion of natural woman-wit, and slave all day for the merest pittance, with little hope of even setting a foot upon the next rung of the ladder.

We spoke of sempstresses who work in factories in Class II. They are comparatively well off, and no doubt there are a few skilled home-workers who also earn good wages—as things go; but as a rule shirt-makers are in a state of abject poverty which cannot well be exaggerated. The makers of these common shirts are divided into two classes, the "machinists" and the "finishers." Machining is the more skilled work of the two, and commands a better price; but the machine has to be hired, or paid for in weekly instalments, and hard indeed must be the struggles of the poor machinist to keep up her payments when work is slack. Mr. Walker, who gave evidence before the Sweating Committee, tells us that the labour of the machinist of blue Harvard shirts is "harder than that of the hand-sewer, and that she is happy if at the end of the week she can earn seven or eight shillings." Another witness (a female machinist) stated that "she was paid sevenpence or eightpence a dozen for machining shirts, and by working early and late could do a dozen and a-half; the weekly instalment for her machine was half-a-crown."

There is a large amount of sub-contract—or sweating—in this trade; and with a view of finding out whether the wages paid by shirt-manufacturers *minus* the profit of the sub-contractor were sufficient for the shirt-makers to live on, Mr. Walker tells us that he obtained work from a manufactory, and for five months employed a dozen or so of women, dividing among them the entire sum paid by the manufacturer. He found that shirt-machinists could just live upon the wage thus obtained, earning from seven to twelve shillings per week; but the case of the poor "finishers" was found to be far more hopeless. "As for the finishers," he says, "the wages are below living point altogether." We hear of women taking shirts to finish at threepence or twopence per dozen, and only finishing a dozen or a dozen and a-half in a day, finding their own thread, and perhaps being forced to spend money in tram fares when they carry their work home. Truly, we could not marvel if others followed the example of the wretched man (the case was lately reported—we think in the daily papers) who lay upon a bed of sickness day after day, week after week, watching his wife and daughters slaving away at the blue-checked shirts, and straining every nerve to support themselves and him, until at last "the pity of it" became too much for him, and he took away his life, that he might be a burden on them no longer.

### Sack Makers.

Those sack-makers who work in manufactories, earning perhaps twelve shillings a week, are far better paid than the home-workers, to whom is entrusted, we may suppose, the work requiring least skill. Sack-making, one would think, can never require a great amount of skill, but it is very hard work, the carrying to-and-fro of tarpaulins and bundles of sacks being in itself a heavy tax upon the strength of a woman. It would be impossible to do this work without a palm-thimble—a flat, round bit of lead or steel, fixed on a broad piece of leather, which is fastened with a buckle and strap round the hand, so that the thimble comes in the middle of the palm. A steel thimble of this kind is so expensive that the poor workers are generally obliged to be content with leaden ones, which get quickly worn, and are apt to let the needle slip off them, making deep and painful wounds in the hand. It has to be mentioned, again, that sack-makers can never sit to do their work; it is only by standing up that they can get force enough to push their needles through the stiff canvas. A good deal of their work is done for Government; and if this industry did not enjoy the distinction of being one of the worst paid in London, who knows but what the British public might complain of the estimates?

### Umbrella Makers.

It is a positive relief to turn to an industry which brings to a certain number of its workers a fair remuneration, and to none the wretched pay of which we have been of late speaking. Umbrella making requires some skill; and here, no doubt, we have the reason why wages are higher in it than in many other industries. The cheapest umbrellas are sent to Africa, China, and India; they are mostly made by low-class home-working Jewesses, who are supplied with the frames and the triangles of material required for the umbrella. Some of these Jewesses are only paid ninepence a dozen; and although they may be able to earn from twelve to twenty shillings in busy times, it is only for six or seven weeks in the year that they can get full work. But the makers of a better class of goods are paid at a much higher rate, and can earn two shillings a-day, or more if they work long hours. Those women who "finish" the very best parasols can probably earn a good deal more still; but all the home-workers in this industry have to reckon upon having slack seasons, and complain bitterly of the time they are obliged to waste in waiting for work at the warehouses.

The manufacture of silk for these umbrellas (and for ties) is also to some extent a woman's industry. Umbrella silk is usually made by girls working in a factory; but in the manufacture of silk for ties, a silk-weaver is generally helped by his wife and daughters, who, if they can give their whole time to it, may earn something like twelve shillings a-week. These silk-weavers are, as is well-known, intelligent, and we might almost say, cultivated people, several grades higher than the workers whose lot we have of late been describing.

*(To be continued.)*

